# A New Pathology in the Simulation of Chaotic Dynamical Systems on Digital Computers

**DOI:** 10.1002/adts.201900125

**Published:** 2019-09-23

**Authors:** Bruce M. Boghosian, Peter V. Coveney, Hongyan Wang

**Affiliations:** ^1^ Department of Mathematics Tufts University Medford MA 02155 USA; ^2^ Centre for Computational Science University College London London WC1H 0AJ UK; ^3^ Computational Science Laboratory Institute for Informatics, Faculty of Science University of Amsterdam Amsterdam 1098XH The Netherlands

**Keywords:** Bernoulli shift, chaos, dynamical systems, floating point arithmetic, pathology

## Abstract

Systematic distortions are uncovered in the statistical properties of chaotic dynamical systems when represented and simulated on digital computers using standard IEEE floating‐point numbers. This is done by studying a model chaotic dynamical system with a single free parameter β, known as the generalized Bernoulli map, many of whose exact properties are known. Much of the structure of the dynamical system is lost in the floating‐point representation. For even integer values of the parameter, the long time behaviour is completely wrong, subsuming the known anomalous behaviour for β = 2. For non‐integer β, relative errors in observables can reach 14%. For odd integer values of β, floating‐point results are more accurate, but still produce relative errors two orders of magnitude larger than those attributable to roundoff. The analysis indicates that the pathology described, which cannot be mitigated by increasing the precision of the floating point numbers, is a representative example of a deeper problem in the computation of expectation values for chaotic systems. The findings sound a warning about the uncritical application of numerical methods in studies of the statistical properties of chaotic dynamical systems, such as are routinely performed throughout computational science, including turbulence and molecular dynamics.

## Graphical abstract

**Floating‐point numbers are integral to numerical computation**, but have known pathologies, including roundoff and loss of precision. A further pathology is described that manifests itself when they are used to represent the statistical properties of chaotic dynamical systems. It is shown that they lead to errors that are neither obvious nor small, and do not disappear as precision is increased.

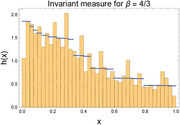

1

Extreme sensitivity to initial conditions is a defining feature of chaotic dynamical systems. Since the first usage of digital computers for computational science, it has been known that loss of precision due to the discrete approximation of real numbers can dramatically alter the dynamics of chaotic systems after a short amount of simulation time. This was observed for the Fermi–Pasta–Ulam–Tsingou problem in the 1950s,^1^ for the Lorenz system and the Hénon‐Heiles problem in the 1960s,^2^, ^3^ for the Chirikov–Taylor map in the 1970s,^4^ and for too many systems to enumerate thereafter. It has long been recognized that extreme sensitivity to initial conditions precludes accuracy of orbits after too long a time, both in the computational and experimental domains. It is well known in turbulence^5^ and in molecular dynamics.^6^

To overcome this problem and restore the predictive power of the scientific method to such systems, dynamicists retreated to the position that, while accuracy for individual orbits may not be possible, accuracy in an averaged sense may still be possible, for some variables of interest in some systems of interest. For example, if the dependent variables of the Lorenz system are denoted by (x,y,z), and if the initial conditions are uniformly distributed in a sphere of unit radius centered at the origin, it may be true that one hundred different computer programs will yield one hundred different answers for (x,y,z) at time 100, but there is hope that the average value of *x* ^2^ is more robust, and that it may be calculated and compared with empirical results. The averaging here may be a time average, an ensemble average, or both.

The entire statistical theory of turbulence is built upon the above supposition. For driven, incompressible Navier–Stokes flow in the turbulent regime, for example, it may be impossible to know the hydrodynamic velocity and the pressure at a particular point in space at a late time, but fluid dynamicists are convinced that it ought to be possible to know, say, the average of the fourth power of the *x* component of velocity divided by its variance squared—a dimensionless number—to very high precision. Lacking any way to compute this number analytically, they routinely resort to digital computer simulation for this purpose. Because the quantity in question is an average over a long time or a large ensemble, they are less concerned about the detrimental effects of floating‐point truncation on individual orbits. There is a vaguely articulated but nonetheless widespread hope that any such errors will not lead to systematic deviations in the statistical quantities of interest. The methods of Direct Numerical Simulation (DNS) and Large–Eddy Simulation (LES) for the Navier–Stokes equations are predicated on this hope, and are routinely used to predict everything from the weather, to the drag past airplanes and automobiles, to the flow of air through ducts and to the flow of blood through our arteries.

Here, we demonstrate that, for at least one simple‐but‐prototypical driven, dissipative dynamical system, namely the generalized Bernoulli Map, the abovementioned hopes are dashed. While it has long been known that individual orbits of this map are chaotic in nature, and that even statistical averages are problematic for the particular case of the system parameter β=2,^7^, ^8^, ^9^ our present work demonstrates a more serious problem. Even assuming generic values of β, and even assuming idealized statistical averages over infinite evolution time and infinite ensemble size, the results of such averages will be inaccurate by factors of order unity. Unlike other sources of error associated with floating‐point numbers, such as loss‐of‐significance errors, noise in function evaluation, and underflow and overflow,^10^ the problem we describe in this work is due to the discrete and finite nature of the floating‐point numbers and the extremely delicate structure of the attracting set of chaotic dynamical systems. Though the root of this problem resides in the use of finite‐precision floating‐point arithmetic, it cannot be mitigated by increasing the precision of the floating‐point representation. Our analysis strongly suggests that the pathology we describe will exhibit for mantissa and exponent fields of any finite length whatsoever, and for floating‐point numbers encoded in any radix whatsoever. Indeed, there is every reason to anticipate that this anomalous behaviour is generic in dissipative chaotic systems of the kind encountered in turbulence and molecular dynamics, and that it is entirely possible that many published results of numerical simulation are substantially inaccurate for this reason.

Single‐precision IEEE floating‐point numbers consist of 32 bits, of the form

$$\left(\sigma ,\;e_{1},\ldots ,e_{8},\;m_{1},\ldots ,m_{23}\right)$$

where σ is the sign bit, the $e_{j}$ for j=1,…,8 are the exponent bits, and the $m_{j}$ for j=1,…,23 are the mantissa bits. The nonzero real number *x* thereby represented is

$$x={\left(-1\right)}^{\sigma }\;{\left(1.m_{1},\ldots ,m_{23}\right)}_{2}\;2^{{\left(e_{1},\ldots ,e_{8}\right)}_{2}-127}$$

where the subscript 2 indicates that the enclosed bits are to be interpreted as base‐2 numbers. For σ=0, as the integer ${\left(e_{1},\ldots ,e_{8}\right)}_{2}$ ranges from 1 to 254,^11^ the format is capable of representing numbers in [1,2) times exponentials ranging between 2^−126^ and 2^+127^. In each interval $\left[2^{j},2^{j+1}\right)$, there are 2^23^ equally spaced single‐precision floating point numbers, distinguished by their mantissa bits. This means that there are as many floating‐point numbers in [1,2) as there are in $\left[\frac{1}{2},1\right)$, as there are in $\left[\frac{1}{4},\frac{1}{2}\right)$, as there are in $\left[\frac{1}{8},\frac{1}{4}\right)$, etc. It is this discrete and uneven distribution of floating‐point numbers, superposed upon the delicate distribution of chaotic attracting sets, that causes the pathology studied in this work. Double‐precision IEEE floating‐point numbers are constructed in similar fashion, but using 52 mantissa bits and 11 exponent bits.

A typical problem in the numerical simulation of chaotic dynamical systems is the estimation of expectation values of observables that depend on the state of the system in a long‐time average, an ensemble average, or both. The effects of the pathology we describe fall into one of three categories, depending on a model parameter: i) observable expectation values that are nearly correct, ii) observable expectation values that are obviously wrong, or, iii) last and most insidiously, observable expectation values that are wrong, but not obviously so. In the last situation, we will demonstrate relative errors of order unity, even though the results might superficially seem accurate.

The model dynamical system that we examine to reveal this new pathology is the *generalized Bernoulli map*, sometimes called the *beta shift*.^12^ Mathematically, this is a dynamical system whose state space is in correspondence with real numbers in the interval [0,1). The initial condition is denoted by *x*_0_. The state of the system at time j+1, denoted by $x_{j+1}$, is given by

$$x_{j+1}=f_{\beta }\left(x_{j}\right):=\beta x_{j}\;\mathrm{mod}\;1$$

For the original Bernoulli map, β was taken to be two, but in this study we examine many different values of β>1. We chose this system because it has a dense and complex attracting set, it is simple enough to examine analytically, an exact expression is known for its invariant measure, and it (or straightforward variants or generalizations of it) is topologically conjugate to many dynamical systems of interest to engineers, biologists, chemists, physicists, and mathematicians. Owing to these properties, we are able to calculate exact expectation values of observables on this set using term‐by‐term integration over the known invariant measure. We refer to the exact value of an observable O(x) calculated in this way as *O*_ex_.

We compare *O*_ex_ with the result that would be obtained by an ideal floating‐point simulation, in which the initial conditions comprise an infinite ensemble randomly sampled from the interval [0,1), each of which is allowed to run for an infinite length of time. To obtain such an idealized result in a finite amount of time using single‐precision floating‐point numbers, we must

1. enumerate all of the limit cycles of the dynamics,
2. identify the basins of attraction of each of those limit cycles in the set of all floating‐point numbers in [0,1),
3. compute the probability that the random number generator will select an initial condition in the basin of attraction of each limit cycle, and
4. average the observable over each of the limit cycles, weighted by their respective probabilities.

In order to do (2) correctly, one has to pay careful attention to the non‐uniform distribution of the floating‐point numbers. The 2^23^ floating point numbers in $\left[2^{-j-1},2^{-j}\right)$ must each be assigned a weight $2^{-j-24}$ so that the total probability of selecting an initial condition in this interval is $2^{23}\times 2^{-j-24}=2^{-j-1}=2^{-j}-2^{-j-1}$, the length of the interval. This may involve combining probabilities of very different magnitudes, and so it is computed by first sorting the list of contributing probabilities and then adding them from smallest to largest, using double‐precision arithmetic, to avoid loss of significance.

We can work this out using single‐precision floating‐point numbers by enumerating all of the asymptotic limit cycles of the dynamics, as well as the fraction of initial states that lie in the basins of attraction of those limit cycles. By computing averages over the limit cycles, and then weighting those averages by the fractional sizes of the corresponding basins of attraction in [0,1), we obtain the result that one would obtain if one could perform an ideal floating‐point simulation of the system, for an infinite period of time and using an infinite number of ensemble elements. We are able to compute this result, which is the best that one can hope for from any floating‐point calculation, only because there are just about a billion^13^ single‐precision floating‐point numbers in [0,1). The structure of the limit cycles thus found permits us to infer how the result would behave for floating‐point numbers of arbitrarily high accuracy, and we call this result *O*_fp_. The relative error between *O*_ex_ and *O*_fp_ is attributable to the newfound pathology.

If β is a positive integer greater than or equal to two, it is easy to understand the effect of the generalized Bernoulli map on the base‐β representation of the state. The action of the map simply eliminates the first digit of $x_{j}$, and shifts the remaining digits one place to the left to obtain $x_{j+1}$. This makes clear that all rational numbers lie on periodic or eventually periodic orbits, since their base‐β digit representations will either repeat or eventually repeat. It likewise makes clear that all irrational numbers lie on chaotic orbits. The state space therefore consists of a dense set of unstable periodic orbits. This set of orbits, and similar sets in more complicated dynamical systems, have been termed “the skeleton of chaos”^14^ because a knowledge of these orbits, including observable averages over them, is sufficient to calculate exact observable averages over the system's invariant measure using the dynamical zeta function formalism.^15^ Unfortunately, as we shall demonstrate, the exquisite complexity of these dynamics is badly damaged by casting it into floating‐point arithmetic.

In spite of the complexity of the generalized Bernoulli map, much is known about it. For any integer value of β≥2, the Perron–Frobenius equation (see, e.g., ref. [16]) can be used to demonstrate that the invariant measure of the dynamics is uniform on [0,1). For non‐integer β, the invariant measure is much more complicated, but an exact expression for it is given by the following series due to Hofbauer^17^

(1) $$h_{\beta }\left(x\right):=C\sum_{j=0}^{\infty }\beta^{-j}\;\theta \left(1_{j}-x\right)$$

where $x_{j}:=f_{\beta }^{j}\left(x\right)$ (so that, in particular, 1_*j*_ denotes $f_{\beta }^{j}\left(1\right)$), θ is the Heaviside function,^18^ and *C* is a normalization constant. Assuming that the orbit ${\left\{1_{j}\right\}}_{j=0}^{\infty }$ is ergodic, the above series makes manifest that the invariant measure has discontinuities at a dense set of points in [0,1). Examples of this invariant measure are shown in **Figure** 1 for three non‐integer values of β, though the reader should be cautioned that these graphs are not as smooth as they appear.

**Figure 1:**
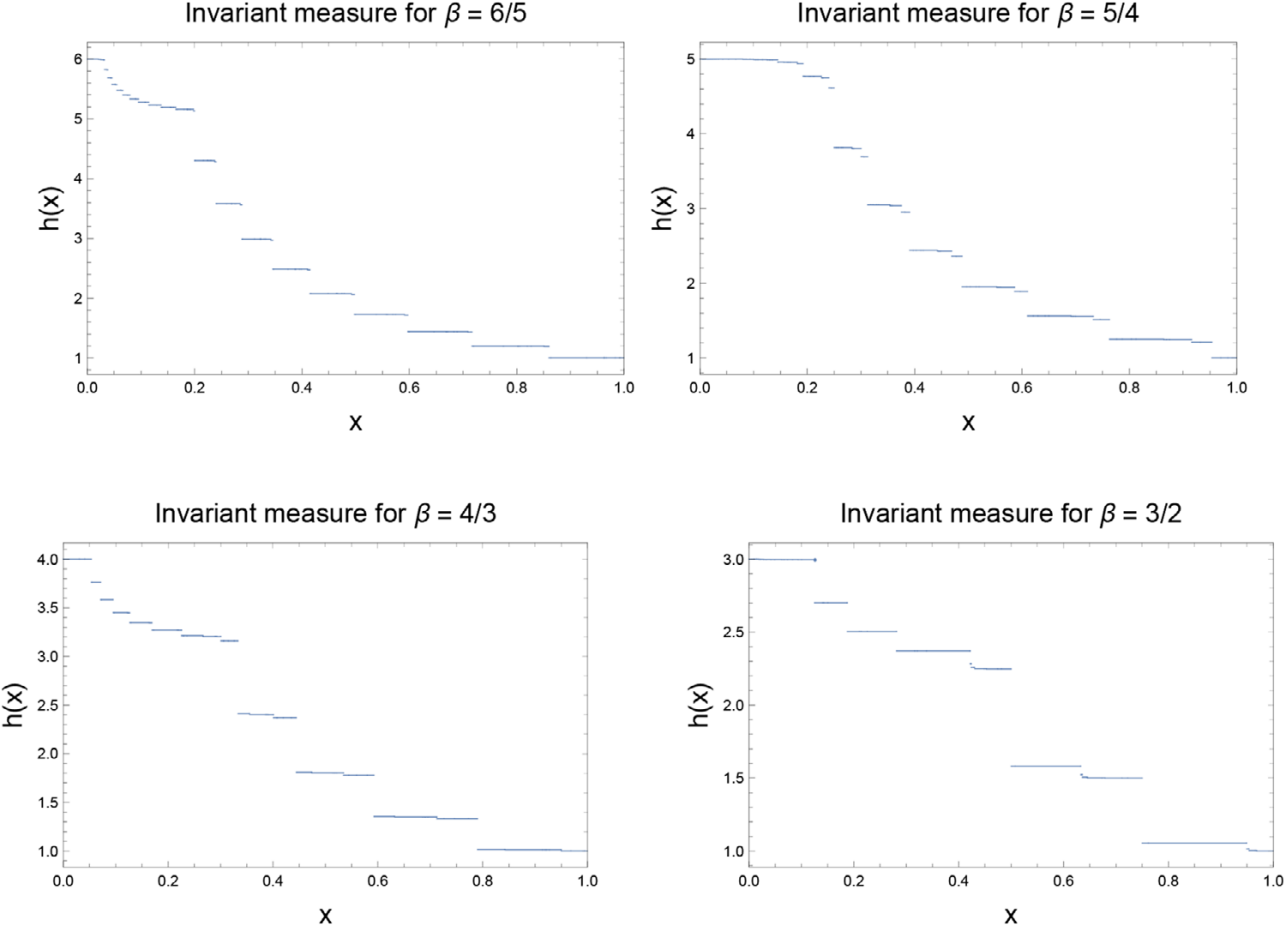
Invariant measures of the generalized Bernoulli map $f_{\beta }$ for $\beta =\frac{6}{5},\;\frac{5}{4},\;\frac{4}{3},\;\frac{3}{2}$. These are normalized so that $h_{\beta }\left(1\right)=1$ (which corresponds to C=1 in Equation (1)). They are not as smooth as these graphs make them appear.

In this study, we use observables of the form $O\left(x\right)=x^{q}$ for q=1,…,100. From Equation (1), it is evident that

O ex =∫01dxhβ(x)xq∫01dxhβ(x)=∑j=0∞β−j∫01dxθ(1j−x)xq∑j=0∞β−j∫01dxθ(1j−x)=∑j=0∞β−j∫01jdxxq∑j=0∞β−j∫01jdx=1q+1∑j=0∞β−j(1j)q+1∑j=0∞β−j(1j)

Because $1_{j}\in \left[0,1\right)$, the error incurred by truncating the above sums can be bounded by a geometric series, allowing us to compute the result to desired accuracy.

The damage that floating‐point arithmetic does to these dynamics is most easily appreciated for the case β=2. Because computer arithmetic is done in base two, and because the binary digits shift one place to the left with each iteration, one bit of precision is lost with each application of the map. Since there are 23 bits of mantissa for single‐precision numbers, the result will be zero after 23 iterations. The use of double precision with 52 bits of mantissa just delays the final result. Either way, the invariant measure will be a Kronecker delta at x=0. If it were possible to let the number of bits of mantissa approach infinity, that Kronecker delta would effectively approach a delta distribution at x=0. The exact time‐asymptotic result for the floating‐point dynamics would never be a uniform measure, the correct answer for the real‐valued dynamics. It is straightforward to demonstrate that exactly the same thing happens for any even integer value of β. Lest the above‐described pathological behavior be dismissed as a consequence of the fact that β is a multiple of two and computer arithmetic is carried out in base two, it may be noted that a computer based on ternary arithmetic would have the same problem if β were any multiple of three, and so on.

It is worthwhile to pause and ask why the spectrum of periodic orbits is damaged to the extent described above. The real Bernoulli map with β=2, for example, has a periodic orbit of period two, wherein $\frac{1}{3}$ maps to $\frac{2}{3}$, which maps back to $\frac{1}{3}$. Unfortunately, the binary expansion of $\frac{1}{3}$ is 0.010101…, and that of $\frac{2}{3}$ is 0.101010…, neither of which terminate. Therefore, no matter how large the number of bits of mantissa, these quantities are not exactly representable as floating‐point numbers, and neither is the periodic orbit that they comprise. Beginning close to a point on this periodic orbit is insufficient because all these orbits are demonstrably unstable. Any roundoff error in the initial condition will inevitably grow. For β=2, this eliminates all orbits except that consisting of the single point {0}. Hence, the time‐asymptotic average of an observable function O(x) will be O fp =O(0), instead of the exact value, which is O ex =∫01dxO(x) because the invariant measure is uniform.

The next line of inquiry that suggests itself is the examination of odd integer values of β. By doing a thorough examination of the periodic orbit spectrum of these dynamical systems for single‐precision arithmetic, we have managed to classify all the periodic orbits of such systems, for any odd integer β. The collection of sets $C=\cup_{i=2}^{\infty }\left\{S_{i}^{+},S_{i}^{-}\right\}$, where $S_{i}^{\pm }:={\left\{\left(2k+1\right)2^{i}\pm 1\right\}}_{k=0}^{\infty }$, partition the set of odd integers greater than or equal to three, and thereby define an equivalence relation ∼ on the odd numbers greater than or equal to three. It is possible to show that equivalent odd values of β have the same periodic orbit spectrum. For odd values of β from 3 to 17, **Table** 1 provides these details. It is seen, for example, that 3 ∼ 11 (both in equivalence class $S_{2}^{-}$), and therefore beta shifts with β=3 and β=11 have the same periodic orbit spectrum. Likewise 5 ∼ 13 (both in equivalence class $S_{2}^{+}$), and therefore beta shifts with β=5 and β=13 have the same periodic orbit spectrum.

**Table 1:** Orbit statistics for odd values of β from 3 to 17, including the class $S_{i}^{\pm }\in C$ to which it belongs, the value of *k* within that set, the number of orbits of various periods, the length *T*_max_ of the longest orbit, and the total number of orbits *N*_orb_

Equivalence class			Periods					Orbit characteristics	
β	$S_{i}^{\pm }$	*k*	2^0^	2^1^	2^2^	2^3^	⋅⋅⋅	*T*_max_	*N*_orb_
3	$S_{2}^{-}$	0	2	3	2	2	2	2^22^	47
5	$S_{2}^{+}$	0	4	2	2	2	2	2^22^	48
7	$S_{3}^{-}$	0	2	7	4	4	4	2^21^	89
9	$S_{3}^{+}$	0	8	4	4	4	4	2^21^	92
11	$S_{2}^{-}$	1	2	3	2	2	2	2^22^	47
13	$S_{2}^{+}$	1	4	2	2	2	2	2^22^	48
15	$S_{4}^{-}$	0	2	15	8	8	8	2^20^	169
17	$S_{4}^{+}$	0	16	8	8	8	8	2^20^	176

Table 1 makes clear that the periodic orbit spectrum for single‐precision floating‐point numbers obtained for odd integer β is very different from that of the real continuum dynamical system. Only orbits consisting of dyadic fractions^19^ can be represented precisely, and these have periods that are restricted to powers of two. The density of orbits as a function of period thus decays exponentially, indicating a negative topological entropy, but the actual topological entropy is easily shown to be lnβ>0. Because knowledge of periodic orbits and their properties is sufficient to work out time‐asymptotic expectation values of observables, one might be concerned that this badly damaged orbit spectrum would lead to correspondingly badly damaged observables.

The exact invariant measure for odd integer β should also be uniform, so that for the observable $O\left(x\right)=x^{q}$ the exact expectation value is O ex =1q+1. **Figure** 2 plots the relative error, (O ex −O fp )/O ex  versus *q*, where 1≤q≤100, for three different odd integer values of β. While the magnitude of this relative error may be regarded as small, it is nonetheless about two orders of magnitude larger than machine precision, and clearly trends upward with *q*.

**Figure 2:**
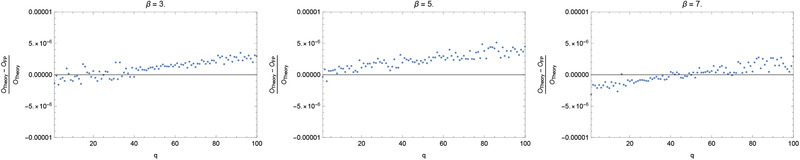
Relative error of the floating‐point calculation of the expectation value of $x^{q}$ for the generalized Bernoulli map $f_{\beta }$ for the odd values β=3,5,7, simulating the average we would obtain if we could run over both an infinite length of time and an infinite ensemble size.

In the more general case of fractional β, the invariant measure has the interesting structure shown in Figure 1. Floating‐point roundoff properties figure necessarily into the calculation of the orbits, and floating‐point orbit periods are no longer restricted to powers of two. The floating‐point orbits themselves are fewer and further between, and orbit periods tend to be much smaller than they are for (odd) integer β. In the case $\beta =\frac{3}{2}$, for example, while there are exact prime periodic orbits for all periods ≥3, there are a total of only ten floating‐point periodic orbits. Beyond the trivial period‐one orbit {0}, the next one has period 186, followed by periods 243, 270, 404, 540, 960, 1800, 3479, and 11050, the last of these being the longest orbit present. It is in such cases that the most serious problems are encountered – serious in that the answers obtained are not obviously wrong, but wrong nonetheless. For observable $x^{q}$, **Figure** 3 plots the relative error, (O ex −O fp )/O ex  versus *q*, where 1≤q≤100, for three different fractional values of β. It is seen that the error incurred can be substantial indeed; for 1≤q≤100, it can reach approximately 2.5% for $\beta =\frac{5}{4}$, 14% for $\beta =\frac{4}{3}$, and 7.5% for $\beta =\frac{3}{2}$. These errors are far greater than those encountered for odd integer β.

**Figure 3:**
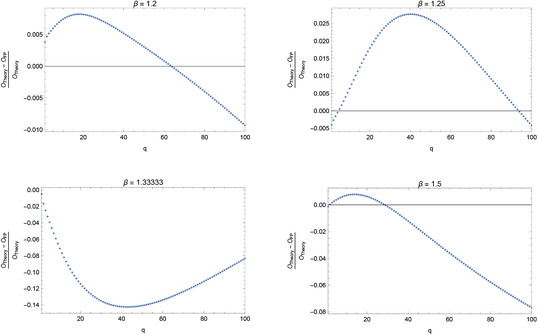
Relative error of the floating‐point calculation of the expectation value of $x^{q}$ for the generalized Bernoulli map $f_{\beta }$ for $\beta =\frac{6}{5},\;\frac{5}{4},\;\frac{4}{3},\;\frac{3}{2}$, simulating the average we would obtain if we could run over both an infinite length of time and an infinite ensemble size.

Thus far, we have restricted attention to observables of the form $x^{q}$. To see that similar problems will be encountered for more general functions of *x*, we can examine the invariant measure itself. The theoretical invariant measure was presented in Figure 1, but we can also compute a “numerical invariant measure.” This is done by taking the collection of all points on periodic orbits, weighting each by the fractional size of the basin of attraction of its orbit, and constructing a weighted histogram accordingly. For even integer β, this would result in a delta distribution at x=0. The histograms for β=3,5,7,9, and for $\beta =\frac{3}{2},\;\frac{4}{3},\;\frac{5}{4},\;\frac{6}{5}$ are presented in **Figure** 4, along with the invariant measures of the corresponding continuum systems, calculated using Equation (1). It is seen that while the invariant measures coincide, at least on the scale illustrated in the figure, for odd integer β, they differ by order unity for non‐integer β. This egregious discrepancy in the invariant measure is the origin of the order unity differences observed between the theoretical and numerical expectation values of $x^{q}$. The above argument makes manifest that similar differences would be observed for the expectation value of almost any function of *x*.

**Figure 4:**
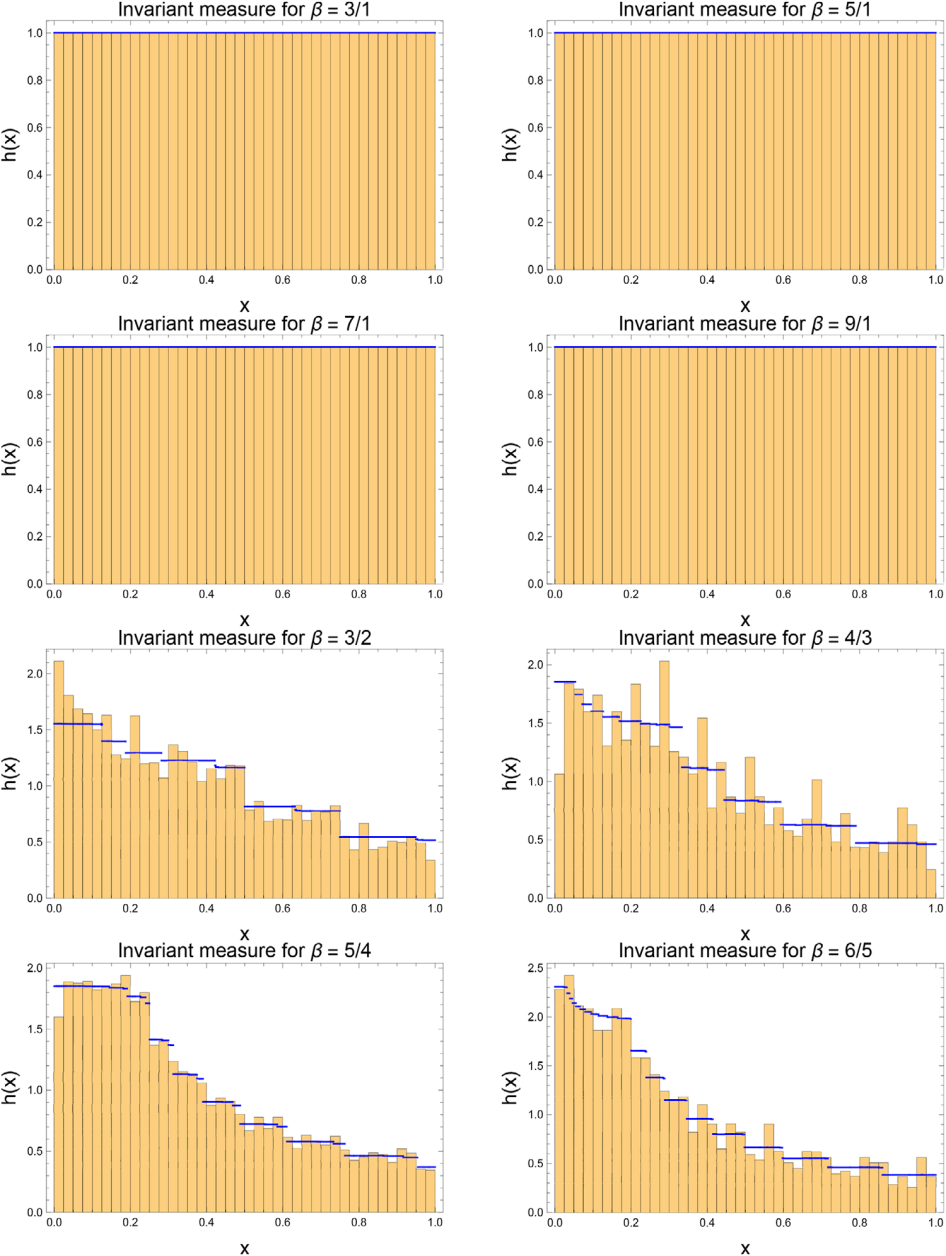
Discrepancy between the exact (blue) and numerical (histogram) invariant measures for the generalized Bernoulli map $f_{\beta }$ for β=3,5,7,9 and for $\beta =\frac{3}{2},\;\frac{4}{3},\;\frac{5}{4},\;\frac{6}{5}$, simulating the average we would obtain if we could run over both an infinite length of time and an infinite ensemble size. While agreement is good for odd integer β (though still greater than roundoff), it is seen to be very poor for non‐integer β.

The suggestion that the error is due to short maximum orbit periods is confirmed by **Figure** 5, which plots the maximum fractional error observed for 1≤q≤100 versus the period of the longest orbit present in the floating‐point dynamics. For the values of β considered, the smallest errors observed were those for $\beta =\frac{6}{5}$ for which the longest orbit period is 36,897. The largest were those for $\beta =\frac{4}{3}$ for which the longest orbit period is only 3,567. The downward trend is clear from the figure. It should be noted that all the maximum periods observed for fractional β are several orders of magnitude smaller than those for (odd) integer β, as recorded in Table 1. This is consistent with the observed discrepancies for fractional β being much higher than those for (odd) integer β. For that matter, it is also consistent with the observed discrepancies for even integer β being highest of all, since those cases have only one orbit of length one, namely {0}.

**Figure 5:**
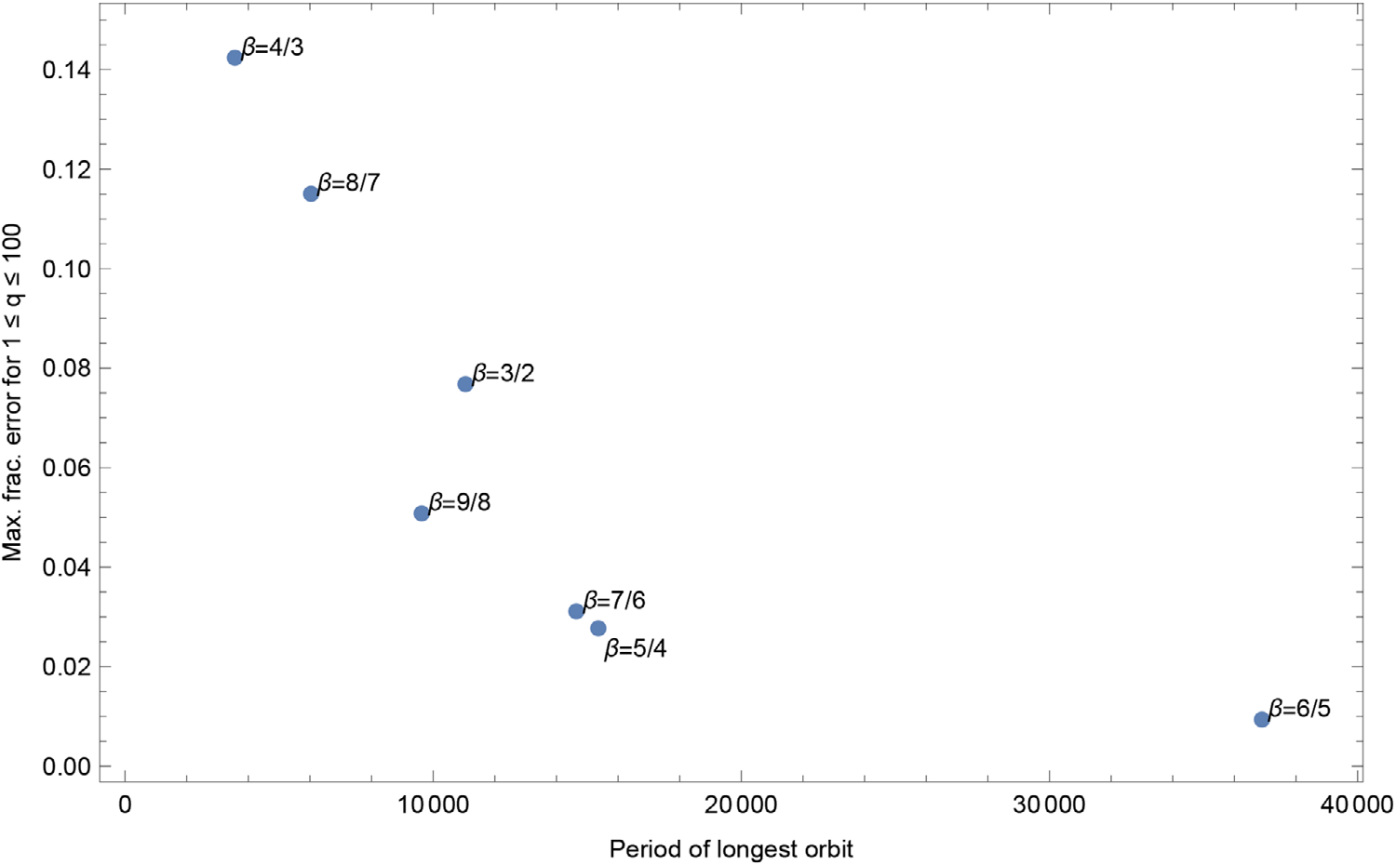
Maximum relative error of the floating‐point calculation of the expectation value of $x^{q}$ for the generalized Bernoulli map for 1≤q≤100, for various values of β, versus the period of the longest orbit present in the floating‐point dynamics.

The grossly truncated nature of the periodic orbit spectrum and the shortness of the orbits for fractional beta strongly suggest that these problems will not be mitigated by increasing the mantissa length. No matter how long the mantissa, floating‐point numbers will always be dyadic rational numbers. The periodic orbit spectrum will always be limited to orbits all of whose states are dyadic rationals. The topological entropy will still be negative, rather than positive. The period of the *k*th floating‐point periodic orbit, ordered by period, is likely to be far smaller than the period of that of the continuum system. In short, simply throwing more bits of precision at this problem is unlikely to make it go away.

Efforts to justify the validity of using floating‐point arithmetic for chaotic dynamical systems often appeal to the *Shadowing Lemma*.^20^ For a discrete hyperbolic map $x_{n+1}=f\left(x_{n}\right)$, this states that for any δ>0, however small, there exists an ε>0 such that a sequence of computer‐generated points ${\left\{y_{n}\right\}}_{n=0}^{\infty }$ for which $y_{n+1}$ is within an epsilon ball of $f(y_{n})$ will itself lie within a δ neighbourhood of some true orbit. If ε is taken to be machine precision, this suggests that a computer‐generated orbit will always be close to an actual orbit. Lest one derive undue comfort from this observation, however, it is important to note that the Shadowing Lemma provides no guarantee that the statistics of the subset of continuum orbits that are shadowed by computer‐generated orbits will be at all similar to those of the entire collection of true orbits of the system.

As an illustration, consider the example of β=2, where the most egregious errors in floating point arithmetic arise. For β=2, the only roundoff error that takes place is when we represent the initial conditions. After that, there is no roundoff error at all. Suppose that we could somehow sample the initial condition *x*_0_ from the true invariant measure on [0,1). The computer will round *x*_0_ to a dyadic fraction *y*_0_, and dyadic fractions all eventually reach the orbit {0} under the action of the map. The sequence reported by the computer, $\left\{y_{n}\right\}$, is an actual orbit; hence it is shadowed by itself. It neither shadows nor is shadowed by the orbit $\left\{x_{n}\right\}$. The sequence $\left\{y_{n}\right\}$ will not remain in any reasonable neighborhood of the sequence $\left\{x_{n}\right\}$ and *vice versa*.^21^ In the end, the sequence $\left\{x_{n}\right\}$ will sample the invariant measure precisely for almost all initial conditions *x*_0_, but the computable sequence $\left\{y_{n}\right\}$ will do nothing of the sort. There is nothing here that is in contradiction with the Shadowing Lemma; the lemma just has nothing to say about the origins of the floating point pathologies we have described.

In conclusion, we have demonstrated a serious systematic error in the numerical calculation of the statistical properties of a very representative chaotic nonlinear dynamical system. This error is distinct from previously studied numerical errors related to rounding and loss of precision, in that it would persist for any finite‐precision mantissa, however large. It arises from the discreteness of floating‐point numbers, their non‐uniform distribution along the real axis, and their inability to represent points on periodic orbits of the dynamics in a precise way, giving rise to a dramatically truncated periodic orbit spectrum. It cannot be mitigated by the use of fixed‐point arithmetic or other recently proposed adjustments to the floating‐point system of representation^22^, ^23^ owing to the discrete nature of any finite‐state digital computer.

It is true that many chaotic dynamical systems of interest in the natural sciences are far more complex than the generalized Bernoulli map presented here. The chaos of turbulent fluid flow, for example, is subtly correlated in ways that have no analogue in the model considered in this paper. We do not believe, however, that practitioners should draw any comfort from the fact that their models are more complex than this one. Rather, we would suggest that if so simple a system exhibits such egregious pathologies, a more complex system will probably exhibit even more devilish ones. Hence we see no reason to doubt that substantial errors of this sort will be present in numerical simulations of chaotic dynamical systems of widespread interest in science and engineering, including computer simulations of thermostatted molecular dynamics,^6^ turbulent fluid dynamics, and reaction–diffusion dynamics, about which until now computational scientists have been completely unaware.

## Conflict of Interest

The authors declare no conflict of interest.

## References

[1] E. Fermi, J. Pasta, S. Ulam, Studies of the nonlinear problems, I, Los Alamos Report LA‐1940 1955; later published in *Collected Papers of Enrico Fermi*, Vol. II, (Ed.: E. Segre), University of Chicago Press, Chicago, IL, USA **1965**, p. 978.

[2] E. N. Lorenz, J. Atmos. Sci. 1963, 20, 130.

[3] M. Hénon, C. Heiles, Astronom. J. 1964, 69, 73.

[4] B. V. Chirikov, Phys. Rep. 1979, 52, 263.

[5] U. U. Frisch, Turbulence: The Legacy of A.N. Kolmogorov, Cambridge University Press, Cambridge, New York 1995.

[6] P. V. Coveney, S. Wan, Phys. Chem. Chem. Phys. 2016, 18, 30236. 27165501 10.1039/c6cp02349e

[7] S. Li, When chaos meets computers, arXiv:nlin/0405038 2005. Submitted on 14 May 2004 (v1), last revised 12 Dec 2005 (v3). https://arxiv.org/abs/nlin/0405038.

[8] S. Li, G. Chen, X. Mou, Int. J. Bifurcation Chaos 2005, 15, 3119.

[9] C. Li, B. Feng, S. Li, J. Kurths, G. Chen, IEEE Trans. Circuits Syst. I: Regular Pap. 2019, 66, 2322.

[10] K. Atkinson, Elementary Numerical Analysis, John Wiley & Sons, Hoboken, NJ, USA 2004.

[11] The values 0 and 255 are reserved for other purposes. The number x=0, for example, is represented by setting both the mantissa and exponent bits equal to zero. Floating‐point numbers between zero and 2^−126^ are called *denormal* numbers, are uniformly spaced in that interval, and are also represented with exponent bits equal to zero.

[12] W. Parry, Acta Math. Acad. Sci. Hung. 1960, 11, 401.

[13] While there are 2^32^, or about four billion single‐precision floating‐point numbers, approximately half of those are negative, and half of those that remain are outside the interval [0,1).

[14] P. Cvitanović, Phys. D 1991, 51, 138.

[15] D. Ruelle, Not. Am. Math. Soc. 2002, 49, 887.

[16] R. C. Robinson, An Introduction to Dynamical Systems: Continuous and Discrete, *Vol. 19*, American Mathematical Society, Providence, RI, USA 2012.

[17] F. Hofbauer, Monatsh. Math. 1978, 85, 189.

[18] For this purpose, we define θ so that θ(0)=1.

[19] Dyadic fractions are fractions with power‐of‐two denominators.

[20] A. Katok, B. Hasselblatt, Introduction to the Modern Theory of Dynamical Systems, Vol. 54, Cambridge University Press, Cambridge, UK 1995, see Theorem 18.1.2. 10.1017/cbo9780511809187.

[21] One could make the neighborhood the entire sequence space, but then the Shadowing Lemma reduces to a triviality.

[22] J. L. Gustafson, The End of Error: Unum Computing, Chapman and Hall/CRC Press, Boca Raton, FL, USA 2015.

[23] J. L. Gustafson, W. Kahan, The Great Debate: John Gustafson and William Kahan, in: Proc. 23rd IEEE Symp. Computer Arithmetic, IEEE, Piscataway, NJ, USA 2016, Special Session: The Great Debate.

